# Clinical applications of concentrated growth factors combined with bone substitutes for alveolar ridge preservation in maxillary molar area: a randomized controlled trial

**DOI:** 10.1186/s40729-021-00396-x

**Published:** 2021-11-29

**Authors:** Shi-chen Lin, Xin Li, Hang Liu, Fang Wu, Lian Yang, Ya Su, Jun Li, Shao-yu Duan

**Affiliations:** 1 Department of Stomatology, Electric Power Teaching Hospital, Capital Medical University, No.1, Taipingqiao Xili, Fengtai District, Beijing, 100073 China; 2 Department of Pathology, Electric Power Teaching Hospital, Capital Medical University, No.1, Taipingqiao Xili, Fengtai District, Beijing, 100073 China; 3 Department of Implant Dentistry, Beijing Stomatological Hospital, Capital Medical University, No.4 Tiantan Xili, Dongcheng District, Beijing, 100050 China

**Keywords:** Alveolar ridge preservation, CGFs, DBBM, Radiography, Histology

## Abstract

**Purpose:**

To evaluate the clinical effects of concentrated growth factors (CGFs) combined with bone substitutes for alveolar ridge preservation (ARP) in the maxillary molar area.

**Methods:**

Thirty-six patients who underwent extraction of the upper molars were recruited and randomly divided into three groups: 1. Grafted with CGFs combined with deproteinized bovine bone mineral (DBBM) and covered with CGFs membrane (CGFs/DBBM group), 2. Grafted with DBBM alone and covered with collagen membrane (DBBM group), 3. Control group spontaneous healing. The area of the alveolar bone in center (C-), mesial (M-) and distal (D-) section was compared with preoperative in radiography. Bone cores were obtained for histopathology observation and comparison.

**Results:**

In C-, M- and D-section, the alveolar ridge area in all three groups was significantly reduced at 8 months postoperatively compared to the baseline (*P* < 0.05). The alveolar ridge area declines in the CGFs/DBBM group (C-12.75 ± 2.22 mm^2^, M-14.69 ± 2.82 mm^2^, D-16.95 ± 4.17 mm^2^) and DBBM group (C-14.08 ± 2.51 mm^2^, M-15.42 ± 3.47 mm^2^, D-16.09 ± 3.97 mm^2^) were non-significant differences. They were significantly less than the decline in the control group (C-45.04 ± 8.38 mm^2^ M-31.98 ± 8.34 mm^2^, D-31.85 ± 8.52 mm^2^) (*P* < 0.05). The percentage of newly formed bone in the CGFs/DBBM group (41.99 ± 12.99%) was significantly greater than that in DBBM group (30.68 ± 10.95%) (*P* < 0.05). The percentage of residual materials in the CGFs/DBBM group (16.19 ± 6.63%) was significantly less than that in the DBBM group (28.35 ± 11.70%) (*P* < 0.05).

**Conclusion:**

Combined application of CGFs and DBBM effectively reduced the resorption of alveolar ridge and resulted in more newly formed bone than the use of DBBM with collagen membranes.

## Background

Dental implants have gained popularity over time and are widely used to restore teeth. The success of implants is directly associated with their osseointegration with the alveolar bone [1]. Therefore, the quality and quantity of a patient’s alveolar bone play important roles in the success of the implants. However, the resorption of the residual alveolar ridge following tooth extraction varies and further, excessive resorption may make implant surgery difficult [2, 3]. It is well established that tooth extraction is followed by a reduction in the buccolingual and apicocoronal dimensions of the alveolar ridge at the edentulous site. Maxillary molars are the most common missing teeth [4] and loss of these teeth is often accompanied by severe resorption of the alveolar bone. In cases of severe alveolar ridge resorption, complex bone augmentation surgery such as maxillary sinus lift is often indicated prior to the implant placement [5]. Maxillary sinus lift procedures not only introduce the complication of unnecessary surgery, but also increase the cost for patients. Occasionally, the use of short implants may avoid complex bone grafting, but may result in an excessive crown to implant ratio and the possibility of bio-mechanical complications [6, 7]. Therefore, it is of great clinical significance to reduce the alveolar bone resorption after tooth extraction and to preserve sufficient bone mass [8]. A variety of biomaterials and biomolecules for preserving the alveolar bone have been investigated. Growth factors are the driving force for tissue regeneration as they regulate many aspects of cellular behavior. CGFs are the third generation of autologous plasma extracts [9]. CGFs contain higher levels of growth factors, platelets, and cytokines than traditional platelet concentrates, such as platelet-rich plasma (PRP) and platelet-rich fibrin (PRF). Due to their strong ability to promote tissue regeneration, CGFs have been widely used in the field of oral implants [10, 11]. In the current randomized controlled trial, a combination of CGFs and DBBM was applied to the extraction sites of maxillary molars to preserve the alveolar ridge. Therefore, the aim of the present study was to evaluate the clinical effects of CGFs combined with DBBM for ARP in the maxillary molar area.

## Methods

### Study design and participants

The present prospective single-center randomized controlled trial was approved by the institutional ethics committee at the Electric Power Teaching Hospital of Capital Medical University, Beijing, China (Approval ref No.: ky-2018-037-02), and the study was conducted in accordance with the Helsinki Declaration of 1975, as revised in 2013. Thirty-six participants who underwent extraction of the upper molars were recruited from the Department of Stomatology, Electric Power Teaching Hospital of Capital Medical University during the period from June 2018 to August 2020.

Informed consent forms were signed by all included participants, and the participants were randomly assigned to three groups: grafted with CGFs combined DBBM and further covered with CGFs membranes (CGFs/DBBM group), grafted with DBBM alone and covered with collagen membranes (DBBM group), Control group with no grafting procedure and spontaneous healing.

The randomized sequence was generated using SPSS 26.0 (IBM, USA) and concealed from the study clinician. The assignments were revealed to the clinician on the day of treatment.

### Inclusion and exclusion criteria

The inclusion criteria were as follows: (1) Presence of upper molars that are not restorable; (2) Preoperative CBCT indicated the presence of at least one adjacent tooth and three bone walls at the extraction site; (3) Periodontal tissues are healthy with no signs of severe periodontal diseases; (4) Signed the informed consent form voluntarily. The exclusion criteria were: (1) Acute periapical periodontitis; (2) Moderate to severe absorption of mesial and distal bone plates; (3) History of local radiotherapy for head and neck regions in the last 5 years; (4) History of using high-dose steroids and drugs that affect bone metabolism; (5) Excessive drinking and smoking (> 10 cigarettes/day); (6) Pregnant or breastfeeding women; (7) Uncontrolled diabetes, serious mental illness, or any other systemic diseases.

### Presurgical treatment

All routine laboratory investigations were assessed before the procedure to avoid further complications during the trial. Clinical examination and supragingival scaling were performed for all of the patients 1 week prior to the surgical procedure. CBCT (KaVo 3D eXam®, KaVo, Germany) scans were taken with a scan time of 8.9 s, 120 kV, 5 mA, and 0.25 mm slice thickness for each patient before surgery.

### Preparation of CGFs

Four 9 ml vacuum tubes without anticoagulants were used to collect the patients’ venous blood, which was immediately processed in the rotating cylinders of a centrifugal accelerator (Medifuge®, Silfradent, Italy). The cylinders were accelerated for 30 s, centrifuged at 2700 rpm for 2 min, 2400 rpm for 4 min, 2700 rpm for 4 min, and 3000 rpm for 3 min, and decelerated for 36 s to stop. The test tube was divided into three layers. The top layer consisted of serum, the middle layer consisted of CGF gel, which was a light yellow gelatin structure, namely the fibrin layer (the main carrier of CGFs) and the bottom layer comprised of red blood cells (RBCs) and platelets. There were a lot of growth factors at the junction between the fibrin layer and the RBCs layer. The fibrin layer and the junction of the fibrin and RBCs layers were reserved in a container with diluted antibiotics for further use.

### Surgical procedure

**Fig. 1:**
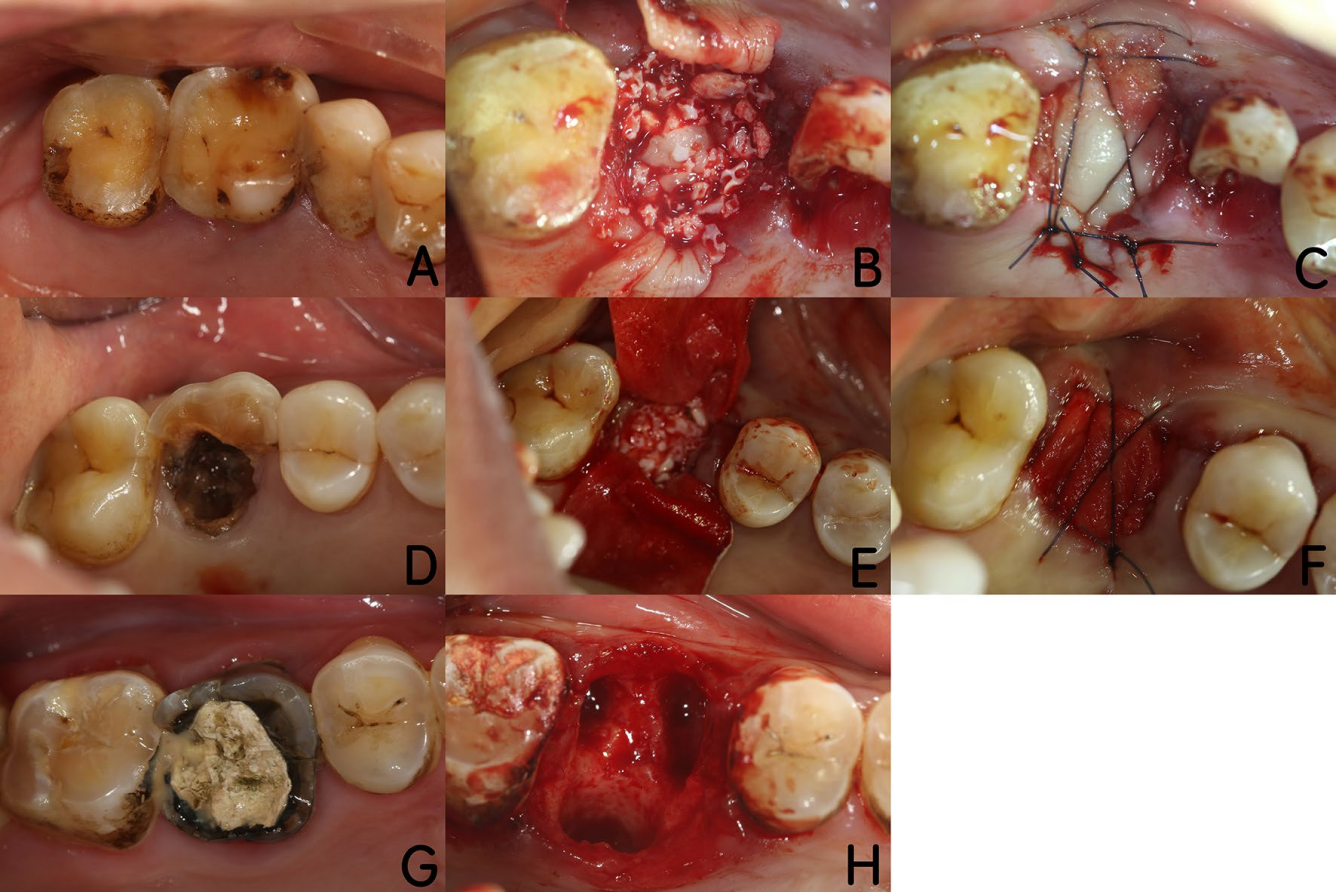
Description of the operative process for the three groups. **a**–**c** CGFs/DBBM group. **a** Preoperative image for CGFs/DBBM group; **b** CGFs/DBBM mixture implanted into the tooth socket; **c** covered with CGF membranes and sutured; **d**-**f**: DBBM group, **d** preoperative image for DBBM group; **e** DBBM implanted into the tooth socket; **f** covered with Bio-Gide® membranes and sutured. **g**–**h** control group, **g** preoperative image for control group; **h** nothing implanted

The surgical procedure was performed under local anesthesia (4% articaine hydrochloride with 1:100,000 epinephrine tartrate). The tooth was extracted using a minimally invasive method and the socket was debrided for complete removal of the inflammatory granulation tissues. Then the patients were randomly assigned to one of three groups according to a concealed randomization envelope. For the CGFs/DBBM group, CGF gels were cut into small pieces and mixed with DBBM (Bio-Oss®, Geistlich, Switzerland) at a 1:1 ratio (volume fraction). Then the mixtures were grafted into the sockets, covered with CGF membranes, and sutured with 4-0 monofilament nylon sutures (Unik, Taiwan, China). For the DBBM group, the sockets were grafted with DBBM alone, covered with collagen membranes (Bio-Gide®, Geistlich, Switzerland), and sutured with 4-0 monofilament nylon sutures. For the control group, the sockets were filled with autologous blood coagulum and left open for spontaneous healing (Fig. 1).

Patients were instructed to take antibiotics (Cefaclor Capsules, North China Pharmaceutical, China) three times a day for 5 days, analgesics (Ibuprofen, Tianjin Smith Kline & French Laboratories. Ltd, China) if necessary, and rinse twice a day with 0.2% Chlorhexidine (South China Pharmaceutical, China). All patients were recalled for check-up and suture removal (CGFs /DBBM and DBBM group) after 7–10 days.

All patients were recalled after 8 months, and a second CBCT scan was performed using the same settings. The implant surgery was performed under local anesthesia (4% articaine hydrochloride with 1:100,000 epinephrine tartrate). An incision was made over the middle of the alveolar crest and the mucoperiosteal flap was elevated for access. A core of bone about 4–6 mm long was obtained (CGFs /DBBM and DBBM group) using a 2.8 or 3.3 mm internal diameter trephine (Helmut Zepf, Germany), immediately placed in 10% neutral buffered formalin, and sent to the Department of Pathology, Electric Power Teaching Hospital of Capital Medical University. Implants with appropriate dimensions were placed using routine processes and postoperative instructions were given to the patients.

### Radiographic analysis

**Fig. 2:**
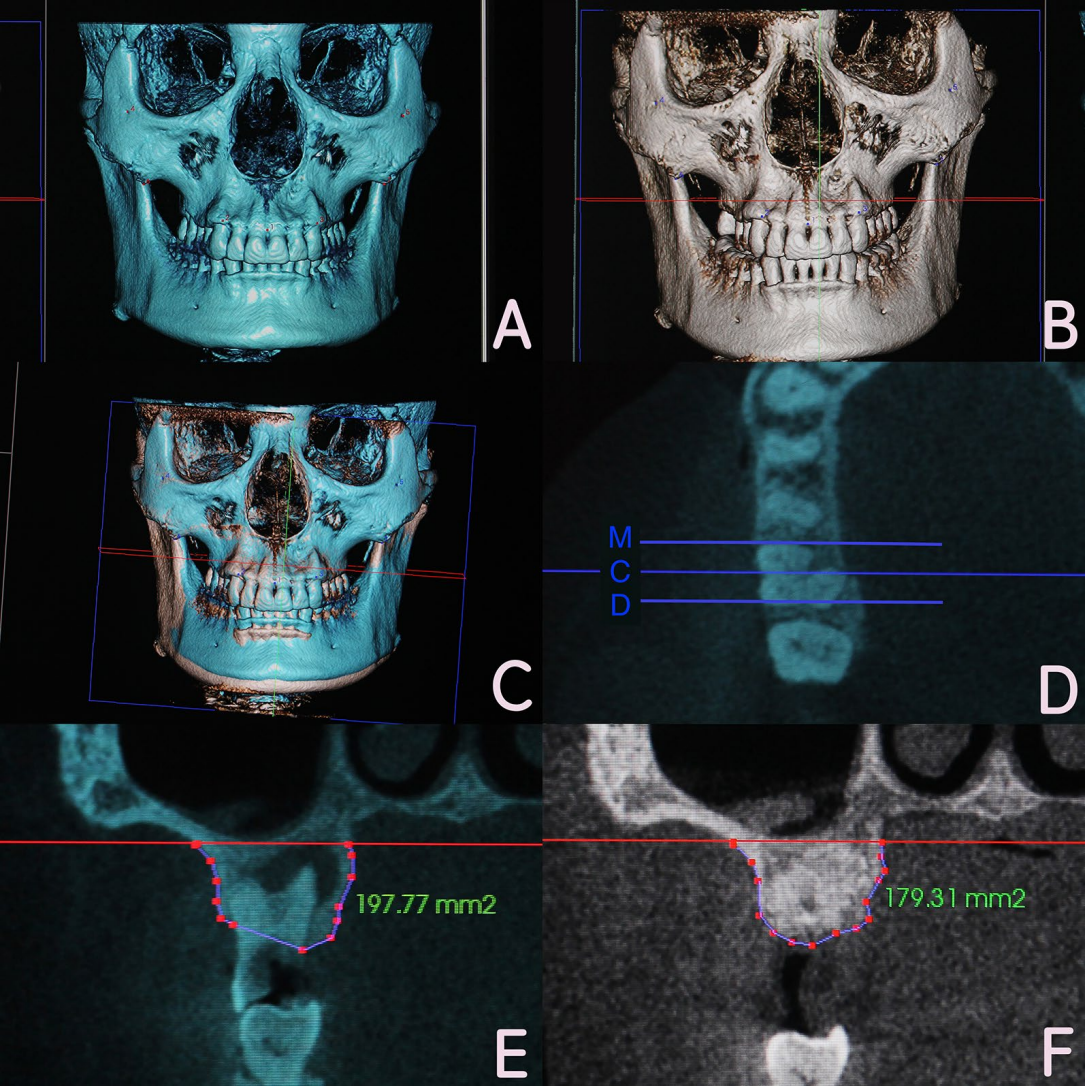
CBCT observation and measurement method. **a** Preoperative three-dimensional image; **b** 8-month postoperative three-dimensional image; **c** three-dimensional image after superimposition; **d** C-, M-, D-section were determined on the superimposed images; **e** the preoperative alveolar bone area was measured; **f** the 8-month postoperative alveolar bone area was measured

The CBCT measurements were performed by an experienced investigator. Pre and postoperative CBCT images from the same patient were superimposed using a 3D image processing software (Invivo 5, Anatomy, USA). Afterward, the superimposed images were transformed to a two-dimensional format. The coronal plane in the center of the extraction site in mesiodistal direction was designated as the C-section. The coronal plane parallel to the C plane and 2 mm from the mesial bone wall was designated as the M-section. The coronal plane parallel to the C plane and 2 mm from the distal bone wall was designated as the D-section. Reference lines were drawn at the bottom of the maxillary sinus on the C-, M-, and D-sections, respectively. Marks were placed along the contour of the alveolar bone under the reference lines in the pre and postoperative images. Alveolar bone areas in each section of pre and postoperative images could be measured by the software, and then recorded for further statistical analysis (Fig. 2).

### Histological analysis

All of the bone cores were decalcified, embedded in paraffin, and cut into 5 μm sections. The sections were stained with hematoxylin and eosin (HE). The images were observed under a light microscope at various magnifications (× 40 and × 100). The total area of the bone core in the × 40  visual field was defined as the region of interest (ROI). The percentage of newly formed bone (NB) and residual materials (RM) in the ROI in CGFs/DBBM and DBBM group were calculated using image analysis software (Image Pro Plus 6.0, Media Cybernetics, USA) for further statistical analysis.

### Statistical analysis

The data were analyzed statistically with SPSS version 26.0 software. The enumeration data were expressed as frequency and percentage. The Fisher exact test was used for comparisons between the groups and the measurement data were expressed as the mean ± standard deviation (Mean ± SD). The Shapiro–Wilk test was used to estimate whether the data followed a normal distribution and homogeneity was assessed with the homogeneity of variance test. If the data followed a normal distribution with the same variance, the *t* test and one-way ANOVA were used to compare the intra-and inter-group parameters, respectively, and the least significant difference (LSD)-*t* test was used for post-hoc test. For non-normal distribution, the Kruskal–Wallis *H* test was used. All the data were measured twice by the same person every 7 days. A *P* value less than 0.05 was considered statistically significant.

## Results

### Patients and clinical outcomes

A total of 36 patients (21 males and 15 females with a mean age of 48 years, range 34–65 years) were followed-up for 8 months. There was no significant difference in age, gender, tooth position, and reason for extraction between the three groups (*P* > 0.05), as shown in Table 1.

**Table 1:** Demographic data of included patients

Characteristics	CGFs/DBBM group (*n* = 12)	DBBM group (*n* = 12)	Control group (*n* = 12)	*P* value
Gender (male/female) (n)	7/5	8/4	6/6	0.911^a^
Age (years, mean ± SD)	48 ± 8	52 ± 7	45 ± 10	0.174^b^
Tooth position (first molar/second molar) (*n*)	8/4	10/2	9/3	0.887^a^
Reason for extraction (residual root and crown/tooth fracture/chronic periapical lesion) (*n*)	6/4/2	5/5/2	6/3/3	0.963^a^

^a^Fisher exact test

^b^One-way ANOVA

No obvious swelling, pain, or inflammation was recorded for all patients at 7–10 days. At the 8-month follow-up, all the wounds were covered with mature keratinized gingiva, and no inflammation was found.

### CBCT evaluation

**Fig. 3:**
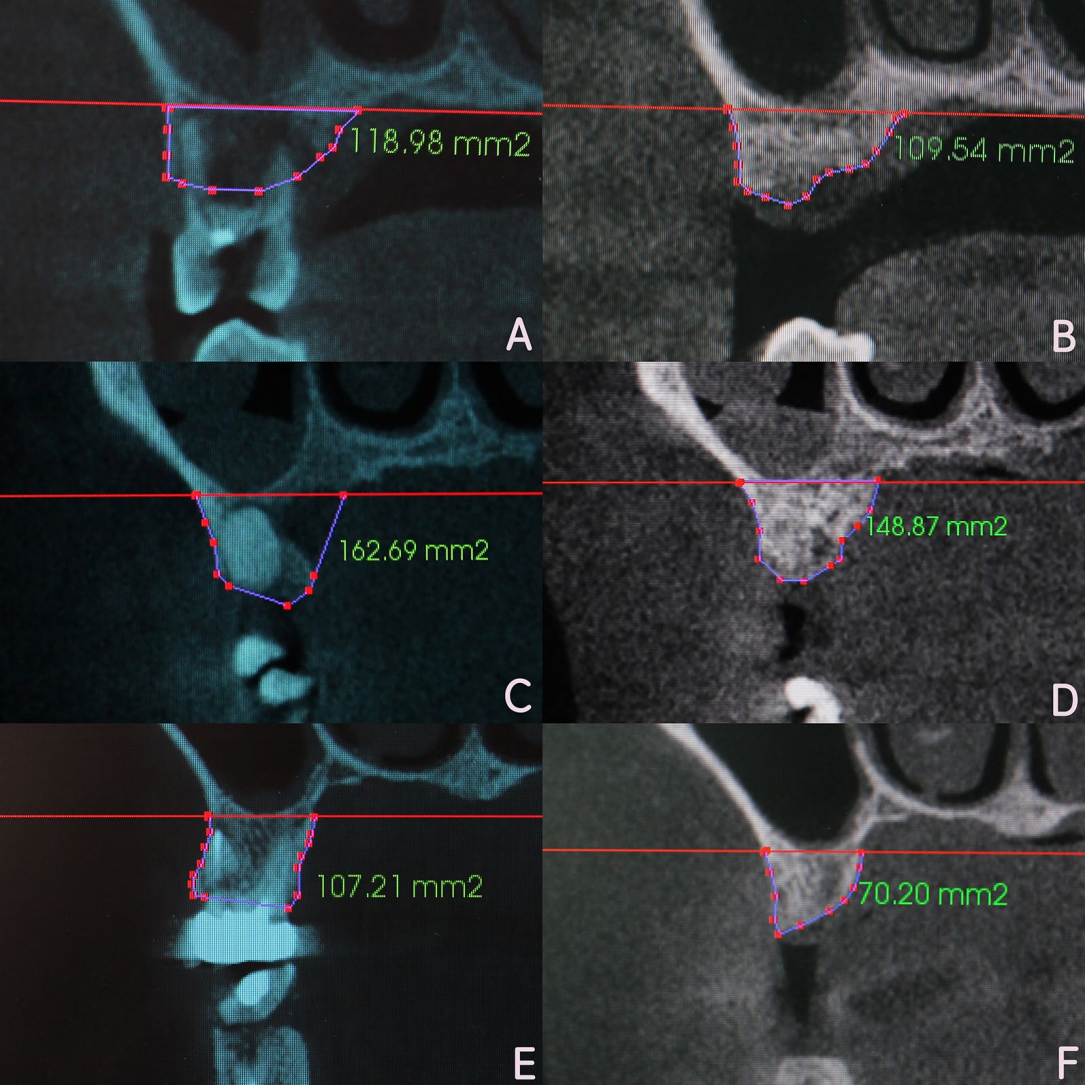
CBCT imaging observation and measurement for the three groups preoperatively and 8 months postoperatively. **a** Preoperative image for CGFs/DBBM group; **b** 8 months postoperatively in CGFs/DBBM group; **c** preoperative image for DBBM group; **d** 8 months postoperatively in DBBM group; **e** preoperative image for control group; **f** 8 months postoperatively in control group;

All the imaging data followed a normal distribution with equal variance. The alveolar ridge area in the C-, M-, D-section for the three groups at 8 months postoperatively was significantly reduced compared to the preoperative value (*P* < 0.05). The alveolar ridge area declines were C-12.75 ± 2.22 mm^2^, M-14.69 ± 2.82 mm^2^, D-16.95 ± 4.17 mm^2^ in CGFs/DBBM group, were C-14.08 ± 2.51 mm^2^, M-15.42 ± 3.47 mm^2^, D-16.09 ± 3.97 mm^2^ in DBBM group, and were C-45.04 ± 8.38 mm^2^ M-31.98 ± 8.34 mm^2^, D-31.85 ± 8.52 mm^2^ in control group. There was non-significant difference between CGFs/DBBM and DBBM group in the three sections (*P* > 0.05). The declines in the two groups were all significantly less than that in control group (*P* < 0.05) (Fig. 3) (Table 2).

**Table 2:** Between and within-group comparison of alveolar bone area in C-, M-, and D-sections for the three groups. (mm^2^, Mean ± SD)

Parameter	Group	Baseline	8 month postoperative	Difference value	*P* value^b^
C-section	CGFs/DBBM (*n* = 12)	144.71 ± 19.54	131.96 ± 18.45	12.75 ± 2.22^†^	< 0.001
	DBBM (*n* = 12)	134.59 ± 21.84	120.51 ± 20.59	14.08 ± 2.51^†^	< 0.001
	Control (*n* = 12)	141.63 ± 24.05	96.59 ± 24.54	45.04 ± 8.38^‡^	< 0.001
*P* value^a^				< 0.001	
M-section	CGFs/DBBM (*n* = 12)	160.96 ± 21.54	146.27 ± 20.23	14.69 ± 2.82^†^	< 0.001
	DBBM (*n* = 12)	146.23 ± 23.71	130.81 ± 24.03	15.42 ± 3.47^†^	< 0.001
	Control (*n* = 12)	139.82 ± 26.07	107.84 ± 22.91	31.98 ± 8.34^‡^	< 0.001
*P* value^a^				< 0.001	
D-section	CGFs/DBBM (*n* = 12)	163.49 ± 22.53	146.54 ± 23.69	16.95 ± 4.17^†^	< 0.001
	DBBM (*n* = 12)	159.38 ± 21.88	143.29 ± 19.21	16.09 ± 3.97^†^	< 0.001
	Control (*n* = 12)	144.71 ± 26.27	112.86 ± 24.26	31.85 ± 8.52^‡^	< 0.001
*P* value^a^				< 0.001	

^a^One-way ANOVA

^b^Paired Student's *t* test

^†^*P* < 0.05 (difference value post hoc LSD-*t* test, statistically significant difference compared with control group)

^‡^P < 0.05 (difference value post hoc LSD-*t* test, statistically significant difference compared with DBBM group)

### Histological observations and histomorphometric measurement

**Fig. 4:**
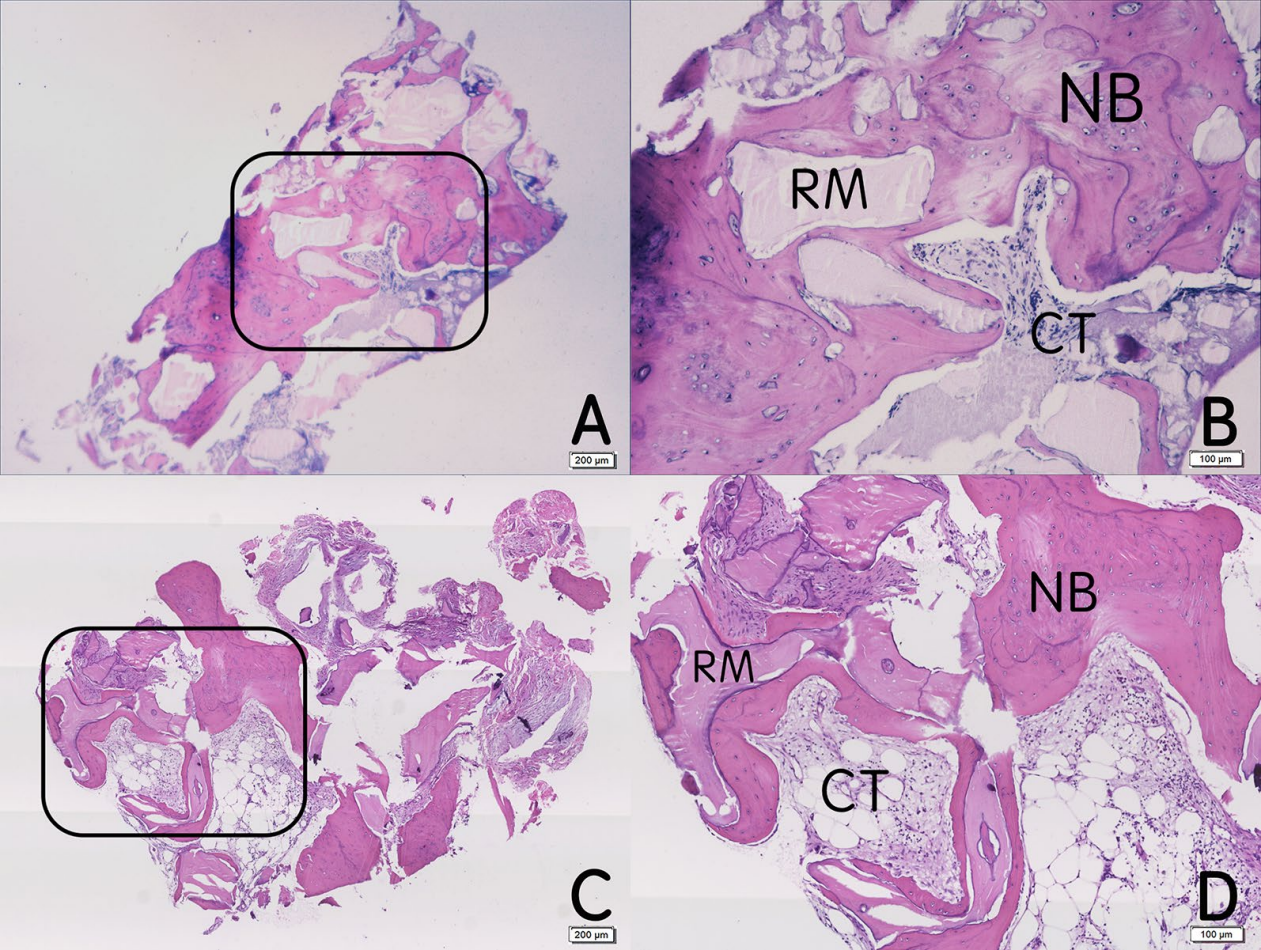
Histological (HE staining) observation of the bone cores 8 months postoperatively for the CGFs/DBBM and DBBM groups. **a** CGFs/DBBM group × 40; **b** CGFs/DBBM group × 100; **c**: DBBM group × 40; **d** DBBM group × 100. *NB* newly formed bone; *RM* residual materials; *CT* connective tissue

In the CGFs/DBBM and DBBM groups, mature lamellar bone formed around the residual DBBM granules. Many osteoblasts were observed at the borderline of the NB, and many osteocytes could be found in the bone lacunae. The percentage of NB in the CGFs/DBBM and DBBM groups was 41.99 ± 12.99% and 30.68 ± 10.95%. There was a significant difference between the two groups (*P* < 0.05). The percentages of RM in the CGFs/DBBM and DBBM groups were 16.19 ± 6.63% and 28.35 ± 11.70%, there was also a significant difference between the two groups (*P* < 0.05) (Fig. 4) (Table 3).

**Table 3:** Comparison of the percentages of NB and RM for the CGFs/DBBM and DBBM groups (%, Mean ± SD)

Parameter	CGFs/DBBM (*n* = 12)	DBBM (*n* = 12)	*t* value	*P* value
Percentage of NB	41.99 ± 12.99	30.68 ± 10.95	2.474	0.031
Percentage of RM	16.19 ± 6.63	28.35 ± 11.70	3.515	0.005

## Discussion

The present randomized controlled trial investigated the clinical effects of combining CGFs and bone substitutes on ARP in the maxillary molar area. Bone graft materials are used frequently [12] and the application of such materials was demonstrated a long time ago. In the 1980s, hydroxyapatite particles were placed in the extraction socket to prevent alveolar ridge resorption [13]. A systematic review and meta-analysis showed that flap elevation, the use of a membrane, and the application of a xenograft or an allograft showed better results in terms of ARP [14]. The first materials used to fill the extraction socket were autogenous bone chips or bone blocks, which are regarded as the "gold standard" for bone graft materials [15]. However, a number of limitations have been noted, such as restricted donor sites, quick absorbance, reports of unpredictable resorption, and pain in some cases. Therefore, a variety of bone substitutes are often used to replace autogenous bone. The present study was performed using DBBM (Bio-Oss®, Geistlich, Switzerland), which has shown promising effects in previous studies [16]. J.R. et al. used three kinds of bone substitutes (Bio-Oss®, Bone Source®, and Embarc®) at the extraction sockets of the premolars in dogs. After 8 weeks, histological observation showed that the untreated and Bio-Oss® sites were similar, with bone filling most of the extraction site. The other two materials did not result in replacement with bone [17]. Cardaropoli et al. noted that Bio-Oss® could significantly slow the resorption of the alveolar ridge and promote the formation of new bone. In addition, combining DBBM with Bio-Gide® membranes could significantly inhibit the vertical and horizontal resorption of alveolar bone [18]. However, researchers have suggested that DBBM lacks osteoinductivity; therefore, a mixture of autogenous bone and DBBM may induce the required osteogenic effects [19]. In the present study, the extraction site was in the maxillary molar area, which contains two or three thick roots and is often associated with varying degrees of bone resorption. Bone removal from different regions such as the maxillary nodule, external oblique line, chin, and other areas for the purpose of obtaining autogenous bone to combine with DBBM may cause additional trauma and discomfort to patients. Therefore, CGFs were mixed with DBBM in the present study instead of autogenous bone.

CGFs contain various growth factors including platelet-derived growth factor [20], transforming growth factor-β, vascular endothelial growth factor [21], insulin-like growth factor, and bone morphogenetic protein [22]. In addition, CGFs have a unique network structure; specifically, fibrinogen molecules form a three-dimensional polymer network that is highly elastic and conducive to the entry of growth factors [23, 24]. Sun et al. preserved the alveolar ridge by implanting CGFs combined with Bio-Oss® into the extraction socket, covered the region with CGFs membranes and sutured tightly. The imaging 6 months later showed that the height and width of the alveolar bone were well preserved [25]. Ge et al. confirmed that CGFs could stimulate the expression of osteogenic genes and promote the osseointegration of implants in animal experiments [26]. In the present study, CBCT images showed that in C-, M-, and D-section, the declines of alveolar area in the CGFs/DBBM group were all significantly less than that in the control group, were similar to those in the DBBM group. It demonstrated that CGFs combined with DBBM could effectively preserve the bone mass of alveolar ridge and provide favorable conditions for later implant surgery.

In histology, the literatures indicated that when grafting was performed with bone substitutes and combined growth factors (CGFs or PRF) in ARP, the healing time was 3–9 months, and the percentage of NB was 37.6–57% [27, 28]. When grafting was performed with bone substitutes alone, the percentage of NB was 11.54–59.5% [29–31]. However, during spontaneous healing without grafting, the percentage of NB was 32.4–41.07% [29, 30, 32]. The reason for the poor osteogenesis of DBBM with collagen membrane might be that these commercial inorganic bone and collagen membranes had biotolerant [33]. They significantly inhibited the alkaline phosphatase (ALP) activity of osteoblasts, increased intracellular reactive oxygen species (ROS), and caused the loss of osteoblast activity and apoptosis [33–35]. CGFs seemed not to be able to reduce the cytotoxicity of bone substitutes. However, it contained a large number of growth factors, which could make synergy and increase ALP activity, promote osteoblast proliferation and differentiation [36, 37]. When CGFs combined with bone substitutes, the mixture possessed osteoinductivity and osteoconductivity. In the present study, CBCT images showed the effects of ARP in the CGFs/DBBM group were similar to those in the DBBM group, yet the histology results indicated the percentage of NB in the CGFs/DBBM group (41.99 ± 12.99%) was significantly greater than that in the DBBM group (30.68 ± 10.95%), and was similar to the proportion of NB in spontaneous healing alveolar ridge reported in the previous literature. It demonstrated that CGFs had a good role in promoting osteogenesis and excellent cytocompatibility, it could increase the proportion of NB and obtain more osseointegration area after placement of implant, be conducive to the long-term prognosis. The reason why the percentage of RM in the CGFs/DBBM group (16.19 ± 6.63%) was significantly less than that in the DBBM group (28.35 ± 11.70%) might be that CGFs and DBBM were mixed in a volume ratio of 1:1, the amount of DBBM implanted into extraction socket was significantly less than that in DBBM group.

Successful ARP requires the use of reliable barrier membranes to seal extraction wounds and promote the growth of hard and soft tissues. At present, the most commonly used barrier membrane is an absorbable collagen membrane [38]. However, most absorbable collagen membranes originate from different species and are a bit expensive. When used in vivo, it showed a continual release of glutaraldehyde with biodegradation and oxidative stress, which led to cell death and dysfunction [35]. As an alternative, CGFs gel is biocompatible and rich in fibrin, contains numerous growth factors, and can be transformed into membranes of the desired thickness using an instrument or gauze. CGFs membranes also have the advantages of toughness and non-immunogenicity [39]. In addition, CGFs membranes can be folded into multiple layers as required to slow degradation and improve the sealing effects in the extraction wound. Fan et al. [40] reported that guided bone regeneration using CGFs as a barrier membrane to repair bone defects in the maxillary anterior teeth showed similar effects to those obtained with the Bio-Gide®. Baniasadi et al. [41] used a demineralized freeze-dried bone allograft combined with PRF and covered in a PRF membrane to preserve the alveolar ridge. The study concluded with satisfactory results. In a similar study by myself and other researchers [42], CGFs were used as a barrier membrane combined with Bio-Oss® for ARP in the maxillary anterior region. We found that the height and width of the alveolar bone were well maintained at 6 months postoperatively. In previous studies, to prevent the loss of bone powder in the tooth extraction socket, some scholars used a periosteal tension-reducing incision to relax the gingival flap and sutured the socket closed, which easily resulted in wound dehiscence and insufficient width of the keratinized gingiva after healing [43]. Other scholars took free gingival flaps from the palatal region and sutured them tightly at the wound site, which caused extra pain to the patients [44]. In the present study, the above shortcomings were avoided. The CGFs membrane could isolate the oral environment, prevent the loss of bone powder, and induce the growth of soft and hard tissues. The results of the current study showed that the wounds of all patients healed well and formed sufficient keratinized gingiva. CBCT imaging data also showed that the alveolar bone was effectively well maintained. It demonstrated that CGFs membrane could achieve better clinical effect and safety than collagen membrane, and be economical.

In terms of analyzing the CBCT imaging data for alveolar ridge evaluation, previous researchers often employed complicated calculations [45, 46] and found it difficult to overlap the same positions accurately in two images. Jung et al. superimposed two CBCT images using an open-source software package (Slicer 3.6, www.slicer.org, USA), which solved this problem [47]. The current study was performed using the Invivo 5 software, which can superimpose two CBCT images very precisely, enabling the surveyor to accurately compare the alveolar ridges in two different images. In addition, the specific method of measurement showed certain advantages in the current study over previous studies in which the alveolar ridge was evaluated by measuring the height and width. As the shape of the alveolar ridge is often irregular, measuring the height and width may lead to inconsistent and inaccurate results. In contrast, in the present study, comparison of the center, mesial, and distal section area in the extraction site facilitated comprehensive and accurate evaluation of changes in the alveolar ridge.

This study was conducted in a very precise and accurate manner. However, there were a few limitations worth noting. The present study results were limited to the maxillary molar region and thus, future research in other regions was needed. Furthermore, the sample size was small and long-term clinical follow-up after implant placement is lacking. The specific mechanism through which CGFs promote the growth of soft and hard tissues is still unclear. Future studies with long follow-up periods and fundamental research on CGF promotion of tissue healing are essential to validate the findings of this study.

## Conclusion

The combination of CGFs and DBBM is a simple and cost-effective method that has been shown to reduce alveolar ridge resorption effectively in clinical applications. Measuring the bone area after superimposing the pre and postoperative images is a simple and accurate method to evaluate changes in the alveolar ridge and can be helpful for further clinical research.

## Data Availability

The data sets generated and analyzed during the present study are available from the corresponding author on reasonable request.

## Abbreviations

CGFs: Concentrate growth factors
ARP: Alveolar ridge preservation
DBBM: Deproteinized bovine bone mineral
CBCT: Cone beam computed tomography
PRP: Platelet-rich plasma
PRF: Platelet rich fibrin
RBCs: Red blood cells
HE: Hematoxylin and eosin
ROI: Region of interest
LSD: Least significant difference
NB: Newly formed bone
RM: Residual materials
